# Enhanced Properties of Collagen Nanofiber Scaffolds via Chitosan/Polypyrrole/Glutaraldehyde Double-Crosslinking

**DOI:** 10.3390/membranes16040129

**Published:** 2026-03-31

**Authors:** Tonantzi Pérez-Moreno, Jesús Humberto Chávez-Meza, Jesús-Salvador Jaime-Ferrer, Gabriel Luna-Bárcenas, Luis G. Arriaga, Janet Ledesma-García

**Affiliations:** 1 Facultad de Ingeniería, División de Investigación y Posgrado, Universidad Autónoma de Querétaro, Santiago de Querétaro 76010, Mexico; tonantzi.perez@uaq.mx (T.P.-M.); jchavez60@alumnos.uaq.mx (J.H.C.-M.); 2 Centro de Innovación Aplicada en Tecnologías Competitivas, CIATEC A.C., Omega 201, Industrial Delta, León 37545, Mexico; jjaime@ciatec.mx; 3 The Institute of Advanced Materials for Sustainable Manufacturing, Tecnológico de Monterrey, Santiago de Querétaro 76130, Mexico; gabriel.luna@tec.mx (G.L.-B.); lg.arriaga@tec.mx (L.G.A.)

**Keywords:** polypyrrole, collagen, nanofibers, glutaraldehyde, double-crosslinking

## Abstract

To enhance the functionality of collagen (Coll)-based scaffolds, we developed a double-crosslinking strategy incorporating an electroconductive chitosan (Ch) and polypyrrole (Ppy) composite. Successful pre-crosslinking of Ch and Ppy was achieved using glutaraldehyde (GTA) at 100 µM. This facilitated imine linkage formation, confirmed by FTIR, enabling synergistic integration with Coll and successful nanofiber scaffold fabrication via electrospinning. While increasing the Ch-Ppy-GTA ratio affected the spinning process and higher GTA concentrations compromised fiber homogeneity, all other measured properties generally improved with increasing ratios. Crucially, this methodology allowed the membranes to maintain their morphology and significantly extended their degradation profile up to 20–30 days in PBS medium at 37 °C. Furthermore, the scaffolds exhibited electroactivity characterized by pseudocapacitance in the presence of Na^+^ and Ca^2+^ ions. These findings demonstrate a robust, tunable method for creating electroactive and structurally stable nanofiber scaffolds suitable for advanced tissue engineering.

## 1. Introduction

Chitosan (Ch), a naturally derived polysaccharide, is widely recognized as a cornerstone biomaterial due to its inherent biocompatibility, biodegradability, and remarkable versatility [1]. Its structure contains free amine and hydroxyl groups, providing abundant reactive sites for chemical modification and crosslinking. These functional groups are critical for tailoring its properties, allowing for control over factors such as mechanical strength and degradation rate, and for integrating advanced functionalities such as electrical conductivity, which is essential for applications targeting electroactive tissues like the heart or brain [2].

To expand the functional scope of chitosan, it is often combined with other polymers or crosslinking agents. Polypyrrole (Ppy), a conductive polymer, is a highly relevant partner for creating electroconductive composite biomaterials [3,4]. However, the successful integration and stabilization of these polymers into a cohesive structure requires efficient crosslinking of both components.

Crosslinking is achieved with various materials, such as PEDOT:PSS [5], genipin [6], EDC, TPP [7] and glutaraldehyde (GTA), which remains favored because of its established efficacy in forming strong, stable bonds and its compatibility with a range of fabrication techniques, from electrospinning to in situ polymerization.

GTA serves as a potent crosslinker due to its bifunctional aldehyde groups, which readily react with primary amines in chitosan [8]. The reaction mechanism typically involves the nucleophilic addition of the amine to the carbonyl carbon of glutaraldehyde, forming an imine (Schiff base) linkage that can be further stabilized through hydrogen bonding or secondary interactions. The degree of crosslinking is influenced by glutaraldehyde concentration, reaction time, and environmental conditions such as pH and temperature [9]. An optimal crosslink density is essential; insufficient crosslinking may result in a scaffold that is mechanically weak and prone to rapid degradation, whereas excessive crosslinking can impede ion diffusion and reduce the overall electrical conductivity of the composite.

Many previous studies using GTA-crosslinked chitosan (Ch) composites have produced non-specific porous hydrogel morphologies, often through GTA vapor treatment or by directly adding the polymers to a GTA solution [10,11,12,13]. However, to effectively mimic the native extracellular matrix (ECM), it is essential to develop scaffolds with defined morphologies, such as fibrous membranes.

Although some groups have successfully reported chitosan and polypyrrole (Ch/Ppy) membranes that retain a fibrous morphology [14,15], challenges remain regarding long-term stability and degradation rate, which are often unreported or limited to a few days. Therefore, the ability to achieve covalent bond formation with low GTA concentrations while preserving fibrous morphology and improving mechanical properties is a critical need in the field.

To address challenges related to the spinnability and inherent structural stiffness of pure Ch/Ppy crosslinked composites, integrating a third component becomes a strategic necessity. Collagen (Coll), the most abundant protein in the ECM, is an ideal candidate [16], providing enhanced biocompatibility and serving as a mechanical reinforcement for the fibers. It imparts greater strength and elasticity, counteracting the inherent fragility of Ppy, and hydrolyzed collagen has been used to prolong degradation [17].

Beyond providing mechanical reinforcement, Coll plays a fundamental bioactive role owing to its close structural similarity to the native extracellular matrix. Recent studies have demonstrated that collagen-based scaffolds are capable of forming well-defined fibrous and mesh-like architectures, which promote cell adhesion, proliferation, and spatial organization, as well as effective integration of the scaffold with the host tissue.

Within this framework, Coll serves as the primary structural and biological component of the scaffold, whereas chitosan, polypyrrole, and glutaraldehyde function as complementary elements designed to enhance its mechanical, morphological, and functional performance.

However, the range of degradation is not always reported; some sources indicate around 7 days [9] when using additional components or GTA vapor, while others report different degradation periods [18]. In this context, GTA concentration is an important parameter because its cytotoxicity can adversely affect cell viability and tissue compatibility. Reported GTA concentrations range from approximately 0.1% [19], 0.5% [20], 4% [8], 20% [9] to 25% [21] in solution or vapor, applied for 2 to 24 h for different tissues.

Crosslinked Ppy-GTA composites have been successfully used in electroactive tissue applications, including injectable formulations for cardiac impulse propagation [22] and scaffolds for neural differentiation via electrical stimulation [23,24]. However, successful clinical advancement of these materials requires a more robust methodology; the lack of detailed mitigation steps highlights the need for stringent washing and subsequent validation of residual compounds [25,26].

Therefore, it is important to ensure that GTA forms covalent bonds at low concentrations, preserves fiber morphology, and enhances mechanical properties. The main objective of this project is to design, fabricate, and characterize electroconductive composite fibrous membranes composed of Ch/Ppy using a pre-crosslinking strategy with GTA at low concentrations. This approach specifically aims to optimize the mechanical, morphological, and degradation properties of the scaffolds to achieve long-term stability (up to 30 days) while maintaining a defined fibrous architecture essential for tissue engineering applications.

## 2. Materials and Methods

### 2.1. Materials

Chitosan (medium molecular weight), glutaraldehyde (GTA), acetic acid, acetone, and ethanol were purchased from Sigma-Aldrich (St. Louis, MO, USA). Phosphate-buffered saline (PBS) and dimethylformamide (DMF) were also obtained from Sigma-Aldrich (St. Louis, MO, USA). Polypyrrole was chemically synthesized following the protocol described by Samwang, T., et al. [27]. Collagen was extracted from bovine tendons [28].

### 2.2. Electrospinning

Chitosan was dissolved in 0.4 M acetic acid, and Ppy was added to the solution at a 1:1 molar ratio with magnetic stirring for 1 h. The solution was then heated to 50 °C, and GTA was added at various molar concentrations determined by stoichiometric analysis (25, 50, 100 µM), followed by magnetic stirring overnight.

Collagen was dissolved in PBS:EtOH as previously reported [29]. Once the solution was prepared, it was mixed with the Ch-Ppy-GTA solutions at a 2:1 molar ratio. Finally, DMF was added to the Coll-Ch-Ppy-GTA mixture at a 1:3 ratio.

The electrospinning conditions for the samples included a voltage range of 18–23 kV, a distance of 12 cm between the syringe and collector, and a flow rate range of 0.1–0.3 mL/h. The rotor collector was set at 100 rpm. The environmental conditions included a humidity range of 25–35% and a controlled temperature range of 30–35 °C, as humidity levels below 25% accelerate solvent evaporation and cause blockages in the flow.

### 2.3. Characterization

#### 2.3.1. Scanning Electron Microscopy

The nanofibers were examined using scanning electron microscopy (SEM) to observe their surface morphology and fiber diameter. An HR-SEM Hitachi SU 8230 (Hitachi High-Tech Corporation, Tokyo, Japan) was used at an accelerating voltage of 1.0 kV and magnifications of approximately ×1000 and ×5000.

#### 2.3.2. Fourier Transform Infrared Spectroscopy (FTIR)

Fourier transform infrared spectroscopy (FTIR) was performed using a Perkin Elmer spectrometer (PerkinElmer, Waltham, MA, USA) with 24 scans at 1 cm^−1^ resolution to determine the chemical composition and integration of the polymers. As a control sample, the Ch-Ppy-GTA solution was dried at room temperature.

#### 2.3.3. Swelling and Weight Loss

Membranes were sectioned into samples of approximately 1 cm^2^ and individually weighed. All tests were performed in triplicate using samples from different membranes. PBS 1X at 37 °C was then added and removed at 1, 5, 10, 15, 20, and 30 days. After these periods, the wet samples were weighed to determine the swelling percentage using Equation (1) [30]:

% Swelling ratio = (W_W_ − W_D_)/W_W_, (1)

where W_D_ is the initial dry weight and W_W_ is the wet weight after each period. In another set of samples, after drying following each period, the weight loss was calculated using Equation (2) [30]:

% Weight loss = (M_i_ − M_f_)/M_i_, (2)

where M_i_ is the initial dry weight and M_f_ is the final weight after each period.

#### 2.3.4. Ionic Conductivity

A type H cell was used to study the transport of Na^+^ and Ca^2+^ ions through the membranes. A 50 mM Tris buffer solution was placed on both sides of the cell, adjusted to pH 7.4, and maintained at 37 °C to simulate physiological conditions. Solutions of NaCl or CaCl_2_ were added to the left chamber to analyze ion passage through the membrane. Electrochemical impedance spectroscopy (EIS) measurements were performed with a 10 mV signal over a frequency range of 0.1 Hz to 1 MHz to evaluate ionic conductivity and membrane performance over 6 days [31]. The ionic conductivity σ was determined using the equation:

σ = l/(R∙A),(3)

#### 2.3.5. Mechanical Properties

Young’s modulus was measured using an AFM NX10 Park Systems (Park Systems Corp., Suwon, Republic of Korea) in PinPoint mode.

## 3. Results

### 3.1. Fourier Transform Infrared Spectroscopy (FTIR)

**Figure 1:**
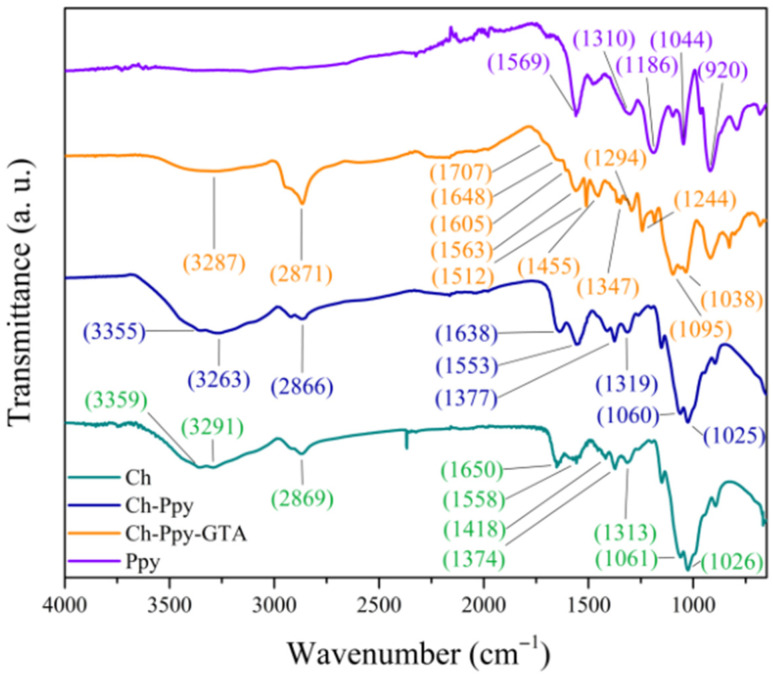
FTIR spectra of Ch and Ppy as controls, the Ch-Ppy mixture, and the crosslinked Ch-Ppy-GTA sample.

The component spectra are shown in Figure 1. The signals related to the Ppy spectrum display representative peaks at 1569 and 1478 cm^−1^ for pyrolytic ring stretching and deformation, 1310 cm^−1^ for C-N and C=C stretching, and 1186 and 1044 cm^−1^ corresponding to C-H in-plane bending and C-H out-of-plane deformation. The signal at 920 cm^−1^ is attributed to biopolaron rings, indicating partially oxidized Ppy [32,33]. For Ch, O-H and N-H stretching are observed at 3359 and 3291 cm^−1^, C-H stretching at 2869 cm^−1^, -CH-OH at 1374 cm^−1^, C-O-C at 1026 cm^−1^ [34], and characteristic signals at 1650, 1558, and 1418 cm^−1^ related to -C=O, NH_2_, and CH_2_, respectively [35].

Additionally, the Ch-Ppy spectrum shows the characteristic signals of both components (Ch and Ppy), but with changes at 1557 cm^−1^, where a left shift indicates tension for amine II stretching, and at 3263 cm^−1^, which is shifted to the right for N-H. These shifts indicate hydrogen bonds between the NH_2_ and OH groups of Ch and the N of Ppy.

For Ch-Ppy crosslinked with GTA, a signal at 3287 cm^−1^ indicates the presence of shifted OH and NH groups. The new signal at 1713 cm^−1^ is related to residual C=O, and 1562 cm^−1^ corresponds to amide II, which is not consumed. In contrast, 1653, 1609, and 1294 cm^−1^ are attributed to the imine group of the Schiff base [9]. Signals at 1510 and 980 cm^−1^ indicate the pyrolytic ring. The signals at 1347, 1244, 1095, and 1038 cm^−1^ are attributed to chitosan. The signal at 1463 cm^−1^ is related to a conformational reorganization of the organic skeleton.

For quantification and confirmation of crosslinking, the difference spectrum shows signals at 1707, 1607, 1575, 1510, and 1469 cm^−1^, which are related to residual C=O, imine groups, amine residues, the presence of Ppy, and -CH_2_ deformation, suggesting partial crosslinking.

**Figure 2:**
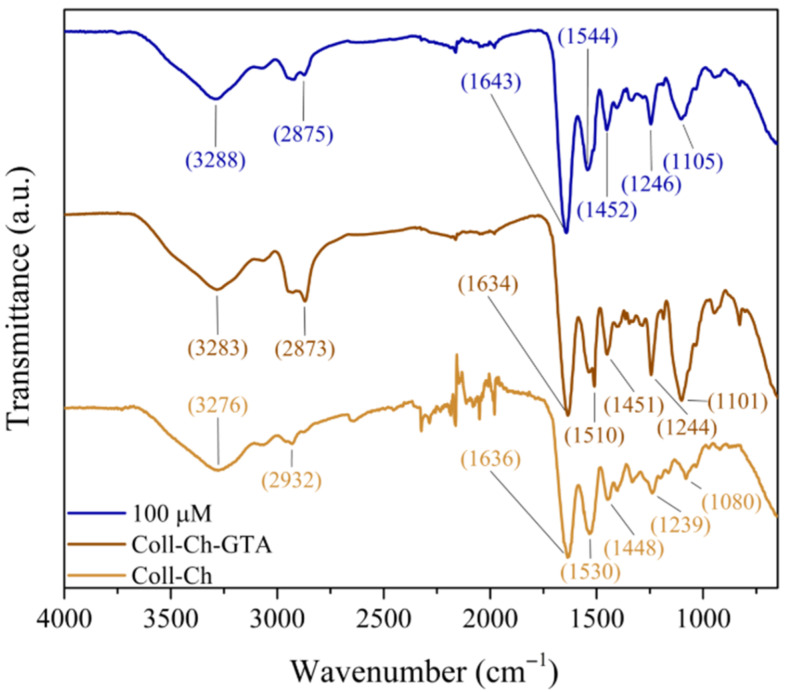
FTIR spectrum of Coll with Ch, crosslinked Coll-Ch with 100 µM GTA, and the membrane with Ppy (Coll/Ch-Ppy-GTA) with 100 µM GTA.

Upon incorporation of Coll, the resulting composite spectra, as shown in Figure 2, display predominant signals characteristic of Coll, notably the major amide groups: Amide I at 1636 cm^−1^, Amide II at 1530 cm^−1^, and Amide III at 1448 cm^−1^ [36]. A distinct double signal between 1537 and 1510 cm^−1^ indicates the presence of both residual NH_2_ and contributions from the Ppy component. The band at 1246 cm^−1^ is consistent with the presence of the imine group formed during the crosslinking process, and at 1105 cm^−1^, the characteristic C–O–C stretching vibrations of the chitosan backbone are observed.

### 3.2. Scanning Electron Microscopy (SEM)

**Figure 3:**
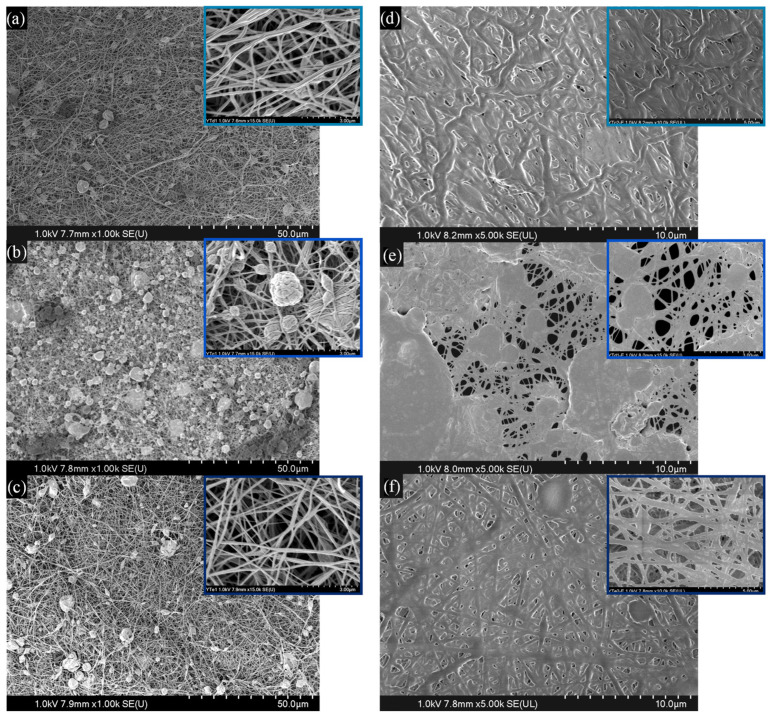
SEM micrographs of Coll/Ch-Ppy-GTA membranes with (**a**) 25 µM, (**b**) 50 µM, and (**c**) 100 µM GTA. Additional vapor of 0.8 µL/mL GTA for 24 h for (**d**) 25 µM, (**e**) 50 µM and (**f**) 100 µM membranes.

Fiber diameter analysis was performed on SEM micrographs by measuring 200 different nanofibers per sample using ImageJ software (v1.54g). As shown in Figure 3, the mean fiber diameters varied with GTA concentration: 101 ± 9 nm for 25 µM, 125 ± 15 nm for 50 µM and 113 ± 15 nm for 100 µM. Figure 3a,b shows representative fiber morphologies; however, in the case of 50 µM, beads are distributed throughout the membrane.

After electrospinning, the membranes underwent an additional crosslinking step involving exposure to GTA vapors. This treatment preserved the nanofiber morphology, with interfiber bonding or fusion at the intersection points of nanofibers [37], as shown in the micrographs in Figure 3d–f; where fiber preservation increased with higher GTA concentrations. This post-treatment, combined with the formation of interwoven fiber bundles during processing, resulted in a noticeable improvement in the mechanical resistance of the final fibrous membranes. However, partial crosslinking left free chains of Coll and Ch susceptible to hydration. Water absorption could lead to swelling and subsequent fusion of these non-crosslinked chains, contributing to the observed collapse or smoothing of the structure upon drying, especially in samples with lower GTA concentrations [9,38].

**Figure 4:**
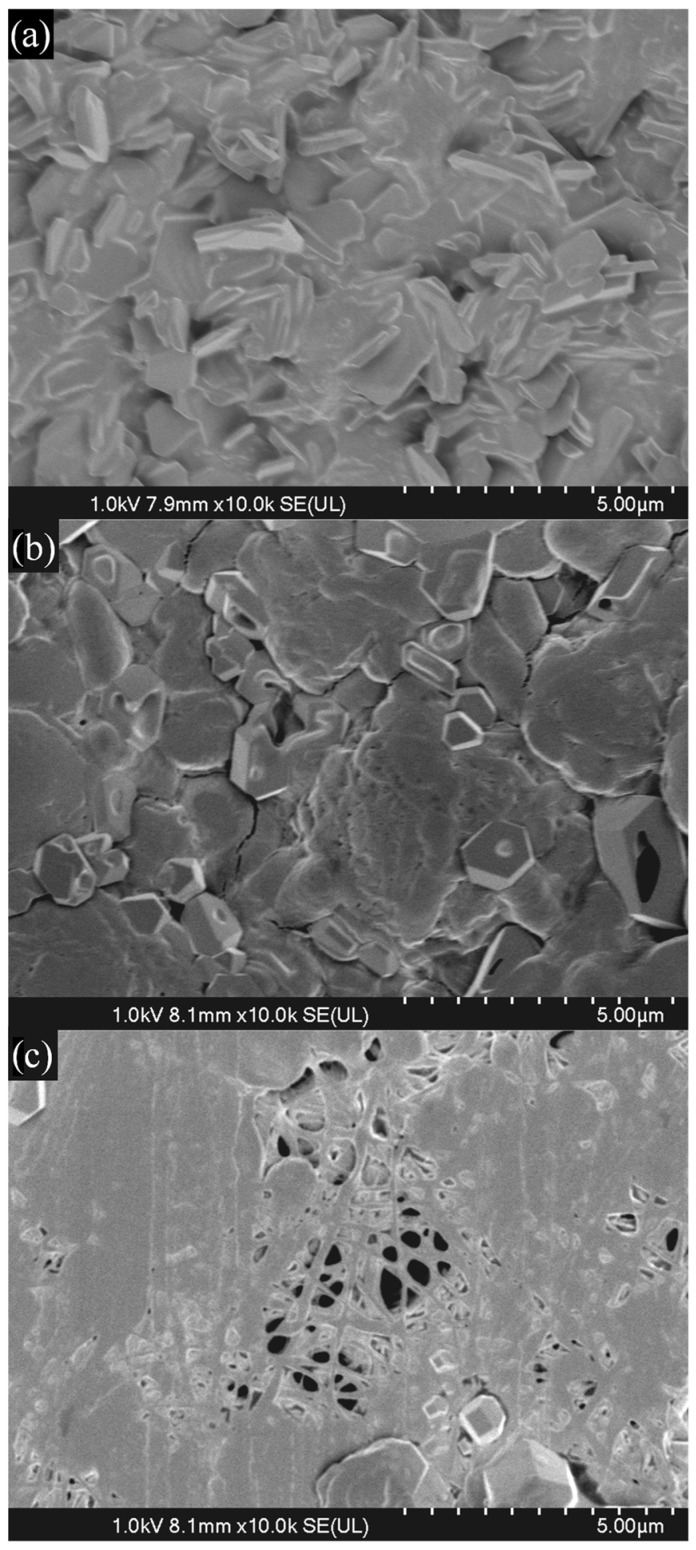
SEM micrographs of membranes with (**a**) 25 µM, (**b**) 50 µM, and (**c**) 100 µM after 10 days in PBS at 37 °C.

SEM analysis of the membranes after 10 days of immersion in PBS solution at 37 °C (Figure 4) revealed a strong correlation between morphological stability and the initial GTA concentration. The structure crosslinked with 25 µM GTA (Figure 4a) showed a clear loss of fiber morphology. The 50 µM sample (Figure 4b) exhibited intermediate stability, losing most individual fiber characteristics but retaining some fibrous conglomerates. In contrast, membranes treated with 100 µM GTA (Figure 4c) largely preserved fiber structure and porosity, confirming the superior long-term structural integrity provided by the highest crosslinking density [39].

### 3.3. Degradation and Swelling

**Figure 5:**
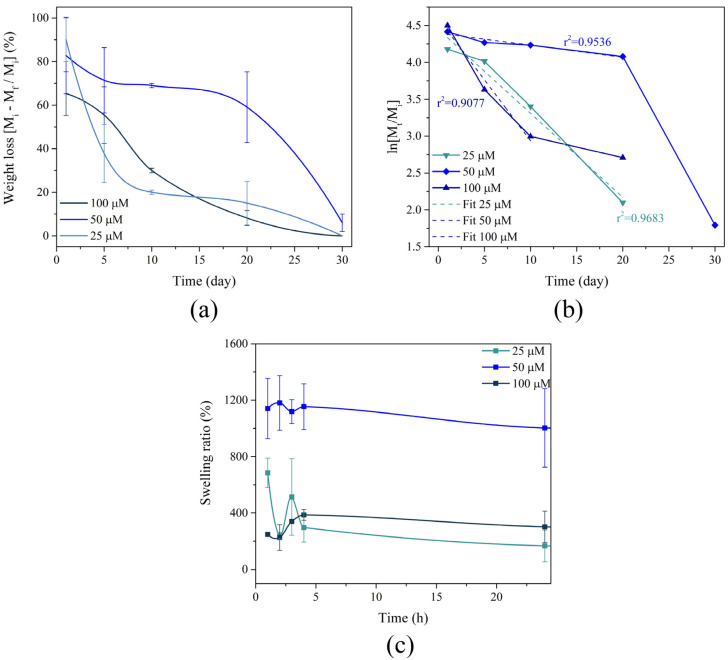
Hydrolytic stability of the crosslinked membranes: (**a**) weight loss, (**b**) kinetic model of weight loss at 5, 10, 20 and 30 days and (**c**) swelling ratio at 1, 2, 3, 4 and 24 h in PBS at 37 °C.

To assess the hydrolytic stability of the scaffolds, the membranes underwent a weight loss assay in PBS at 37 °C (Figure 5). Figure 5a shows a direct dependence on the crosslinking agent concentration: on day 1, membranes with a 100 µM concentration retained 90% of their initial mass, compared to 65% for the 25 µM samples. This difference became more pronounced by day 10, with mass retention at 70% and 30%, respectively. By day 30, both groups of membranes converged, each maintaining less than 5% of their initial mass.

The mass loss kinetics were analyzed by fitting the data to a first-order kinetic model (Figure 5c), which clarified the dominant degradation phases influenced by GTA concentration. For the 50 µM samples, the initial mass loss in the first 10 days is characteristic of surface erosion, primarily attributed to the leaching and hydrolysis of non-crosslinked components [40]. In contrast, the 25 µM samples showed a rapid and sustained mass drop from the onset, suggesting that the insufficient crosslinking density led to early hydrolysis of the few imine bonds present, resulting in rapid structural collapse and fragment release [41]. In the 100 µM samples, stability was maximized: non-crosslinked components were rapidly lost initially, but the high crosslinking density enabled the polymer core to resist bulk hydrolysis for a significantly longer period, validating the superior stabilizing effect of the maximum GTA concentration [42].

The swelling behavior of the membranes was evaluated by quantifying water absorption relative to the dry weight. During the first hour, the membranes exhibited substantial water uptake, absorbing 700, 1190, and 250 times their initial weight in water for GTA concentrations of 25, 50, and 100 µM, respectively. The 25 µM sample showed decreased water absorption, which could be attributed to weight loss due to insufficient crosslinking density [43,44]. In contrast, the 100 µM sample showed increased absorption during the first 4 h but remained below 400% due to high crosslinking density [45]. For the 50 µM sample, water absorption remained around 1200 times the initial weight during the first 24 h.

### 3.4. Ionic Conductivity

**Figure 6:**
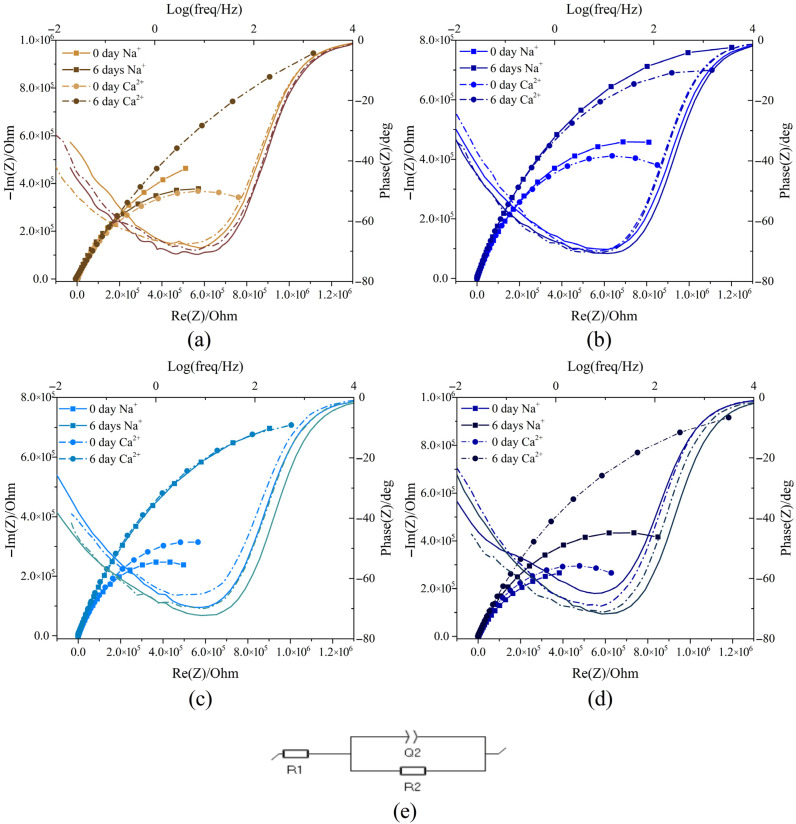
Nyquist plots and Bode phase angle plot of Coll-Ch membranes: (**a**) Coll-Ch, (**b**) 25 µM, (**c**) 50 µM, and (**d**) 100 µM. Measurements were taken at 0 h and 144 h with Na^+^ ions from NaCl and Ca^2+^ ions from CaCl_2_. (**e**) Electronic equivalent circuit of the system.

Ionic conductivity was evaluated using electrochemical impedance spectroscopy (EIS), and the resulting data are presented as Nyquist plots in Figure 6. For both the control membrane and the Ppy-crosslinked membranes, the initial intercept of the high-frequency semicircle with the real axis (Z′) represents the ohmic resistance (R_A_), which is primarily attributed to the electrolyte resistance [46].

Comparing resistance values between day 1 and day 6, a significant increase in resistance was observed. On the first day, resistance ranged from 3.0 × 10^5^ Ω to 4.0 × 10^5^ Ω. By day 6, membrane resistance increased to approximately 8.0 × 10^5^ Ω and 1.0 × 10^6^ Ω for both Na^+^ and Ca^2+^ ions, as shown in Figure 6b,c.

A significant difference in resistance between the two ions was observed only in the control membrane and the 100 µM GTA membrane (Figure 6a,d) when tested with Ca^2+^ compared to Na^+^. This differential behavior suggests selective ion interaction or transport inhibition within the membrane structure [47], potentially due to the higher charge density or divalent nature of the Ca^2+^ ion, which interacts preferentially with the crosslinked polymer network [48].

Conversely, analysis of the Bode phase angle plot suggests a pseudocapacitive behavior within the system (Figure 6e). Specifically, the equivalent circuit’s initial resistance, R_A_, is evident in the high-frequency region, where the phase angle is close to 0°. As the frequency decreases, the slope of the phase curve changes, indicating a transition from resistive control to capacitive or pseudocapacitive control [49].

**Figure 7:**
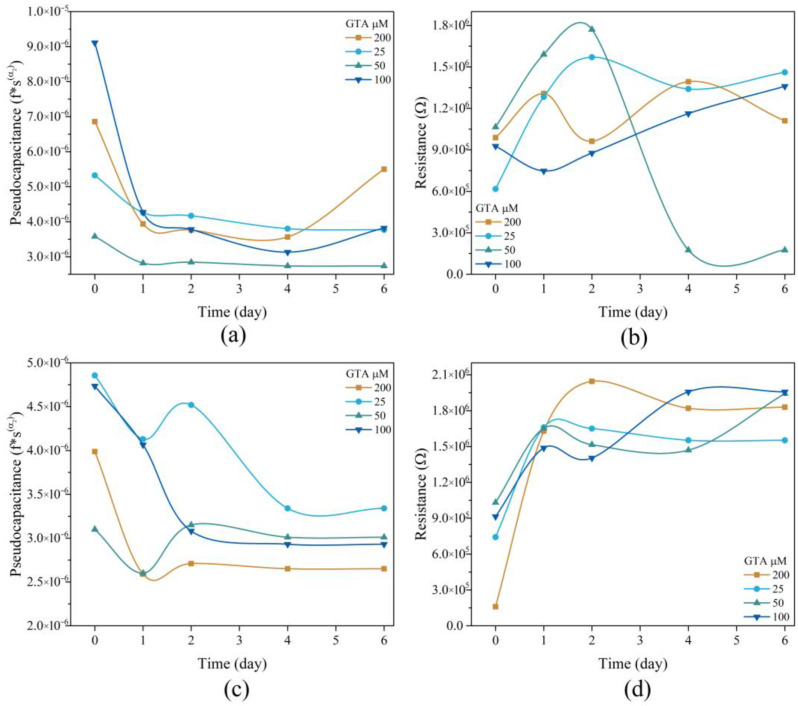
Pseudocapacitance of Coll-Ch, 25 µM, 50 µM, and 100 µM membranes in (**a**) Na^+^ ions and (**c**) Ca^2+^ ions, and resistance (RB) of Coll-Ch, 25 µM, 50 µM, and 100 µM membranes in (**b**) Na^+^ ions and (**d**) Ca^2+^ ions at 0, 1, 2, 3, 4, 5 and 6 days.

Analysis of membrane resistance over multiple days (Figure 7) confirmed distinctive pseudocapacitor behavior. For Na^+^ ions (Figure 7a), membranes with the lowest GTA concentration maintained a relatively constant charge storage capacity over the six-day period. In contrast, when exposed to Ca^2+^ ions (Figure 7c), the capacity varied significantly over time, with the membranes tending to release ions during this interval, suggesting differences in the stability or reversibility of divalent cation interactions [50]. Despite these ionic differences, a consistent trend was observed across all samples: increasing GTA concentration reduced pseudocapacitance capacity from day 1 to day 6 for both Na^+^ and Ca^2+^ ions, likely due to restricted ion mobility from higher crosslinking density [51,52].

Analysis of the final resistance (R_B_) of the circuit (Figure 7b,d) revealed distinct time-dependent behaviors for the two ionic species. With Na^+^ ions (Figure 7b), R_B_ generally increased over six days, rising from an initial 6.0 × 10^5^ Ω to 1.8 × 10^6^ Ω, except for the 50 µM GTA membrane, which showed a substantial decrease in resistance to 2.0 × 10^5^ Ω. For Ca^2+^ ions (Figure 7d), resistance was comparatively lower at day 0, increased sharply by day 1, and then stabilized within the range of 1.5 × 10^6^ Ω to 2.1 × 10^6^ Ω.

**Figure 8:**
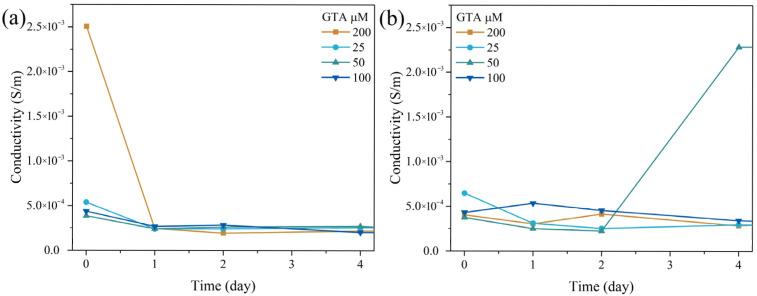
Conductivity of Coll-Ch membranes at 25 µM, 50 µM, 100 µM, and 200 µM as a control for Coll-Ch-GTA in the presence of (**a**) Na^+^ ions and (**b**) Ca^2+^ ions at 0, 1, 2, 3, 4, 5, and 6 days.

The final ionic conductivities (σ) calculated from the impedance data are shown in Figure 8. Using the membrane dimensions (10 mm^2^ area and 400 µm thickness) and the measured resistance values (R_A_, R_B_), the Ppy-crosslinked membranes generally exhibited a conductivity of approximately 5.0 × 10^−4^ S/m with both Na^+^ and Ca^2+^ ions over six days. This conductivity is within the range required for conductive tissue scaffolds, supporting their application in electroactive tissue engineering and validating the use of these membranes for prolonged electrochemical evaluation in such tissues [53].

The specific samples that showed lower resistance in the R_B_ analysis (as mentioned previously) were the only exceptions to this general conductivity, exhibiting predictably higher conductivity of around 2.5 × 10^−3^ S/m. This kinetic shift is directly correlated with the ongoing degradation profile (as shown in Figure 5). The hydrolysis of non-crosslinked components could lead to the formation of wider pathways and increased porosity. This structural relaxation effectively reduces resistance and enhances Ca^2+^ diffusion.

### 3.5. Young’s Modulus

The mechanical assessment involved analyzing the Young’s modulus for the 50 µM GTA membrane and the Coll-Ch-GTA control membrane. The results show that incorporating and crosslinking Ch-Ppy with GTA resulted in a tenfold increase in stiffness compared to the Coll/Ch-GTA control, confirming a substantial enhancement of the membranes’ mechanical integrity. In this case, the Young’s modulus of both membranes exceeds the values required for myocardial tissue, which range from 0.2 to 0.5 MPa [54].

## 4. Discussion

The double-crosslinking strategy in this study was specifically designed to maximize the seamless integration of the electroactive phase Ch-Ppy with the structural component Coll, a critical requirement for advanced tissue engineering scaffolds.

FTIR analysis confirmed the successful formation of Ch-Ppy pre-crosslinking via imine linkages, using GTA concentrations within non-cytotoxic ranges of 100 µM. The subsequent addition of Coll enabled successful electrospinning of scaffolds, producing consistent nanofiber diameters of 101 nm to 125 nm that effectively mimic the native extracellular matrix (ECM).

Post-fabrication stability was significantly improved by the crosslinking methodology. SEM micrographs showed that membranes treated with GTA vapor and those prepared with 100 µM GTA substantially maintained their original morphology and porosity after 10 days of immersion in PBS, while low-crosslinked samples rapidly disintegrated. This direct correlation between crosslinking density and morphological stability was also reflected in the weight loss results. High-concentration membranes retained up to 70% of their initial weight at day 10, extending the practical lifespan of the scaffolds up to 30 days.

The loss of morphological integrity and subsequent weight loss observed in the low GTA concentration membranes is primarily attributed to the hydrolysis of collagen chains and, to a lesser extent, the Ch-Ppy components, which remained available for degradation due to incomplete crosslinking [55,56]. This finding addresses a major limitation of natural polymer scaffolds and is essential for ensuring the feasibility of long-term biomedical applications.

The initial swelling behavior of the membranes was robust, absorbing up to 1200 times their dry weight, which is essential for facilitating nutrient transport and cell adhesion. The reduction or stabilization of the swelling ratio in the first 24 h coincided with progressive weight loss, confirming that structural degradation becomes the primary limiting factor in the long-term water retention capacity of the scaffold, as well as the crosslinking density, which decreased as crosslinking increased. At lower GTA concentrations, the membranes behave as a hydrogel-like matrix [57]; however, the higher number of free functional groups leads to lower resistance and higher pseudocapacitance, compared to higher GTA concentrations, where these sites are consumed by crosslinking, directly affecting ion diffusion.

The pseudocapacitance results indicated more stable and constant ion storage for the monovalent Na^+^ ion, particularly in scaffolds with lower GTA concentrations. This suggests facile ion diffusion through the channels and pores formed by the swollen polymers. In contrast, the Ca^2+^ ion exhibited a more dynamic and less stable interaction, likely due to preferential electrostatic interactions with the functional groups of the Ch-Ppy and Coll network, using amine or hydroxyl groups as charge storage sites, potentially leading to restricted mobility [58].

Notably, although higher GTA crosslinking density provides superior morphological stability, it consistently decreases pseudocapacitance and generally increases final resistance compared to day 0. This structural-functional compromise suggests that high crosslinking density restricts polymer chain mobility and limits ion accessibility to the Ppy and Ch interface, resulting in lower ionic conductivity and reduced charge storage capacity.

Furthermore, the increase in overall resistance observed by day 6 for both ions suggests that ongoing degradation and structural collapse of the non-crosslinked polymer chains within the scaffold matrix gradually restrict the open pathways necessary for efficient ion movement.

Specifically, the ability of calcium ions to form strong coordination bonds with functional groups, such as the amines of Ch or Coll, leads to subsequent inhibition of ion transport [59]. This results in a slower release of Ca^2+^ ions and a greater observed change in resistance compared to Na^+^.

The observed temporary storage capacity of Ca^2+^ ions suggests that the membrane could significantly influence the cell-scaffold interface, potentially promoting or regulating specific signaling pathways critical in cells such as myocytes or osteoblasts for processes including migration, survival, and differentiation.

## 5. Conclusions

This study successfully employed a pre-crosslinking strategy between Ch and Ppy at low GTA concentrations before membrane fabrication, using a double-crosslinking methodology specifically targeting the Coll/Ch-Ppy composite. This critical step enabled effective electrospinning of the composite when combined with Coll. GTA concentration was the key factor in tuning the final membrane properties. This pre-crosslinking method produced robust fibrous membranes that significantly extended degradation profiles, maintaining structural morphology for up to 20 to 30 days—a crucial achievement for long-term clinical relevance.

The membranes showed that increased structural stability from higher crosslinking density led to an inverse relationship with electroactive performance, resulting in decreased pseudocapacitance and increased ionic resistance. This finding underscores that high crosslinking density restricts polymer chain mobility and optimal ion accessibility. Importantly, the optimized low GTA ratio provided the best balance, allowing retention of free functional groups essential for maximal electrochemical performance, even though the highest GTA concentrations compromised fiber homogeneity. Ultimately, this methodology offers a tunable platform for creating functional biomaterials by balancing long-term structural durability with maximal electrochemical performance in scaffolds for advanced tissue engineering.

## Data Availability

Data will be made available upon request to the corresponding author.
